# Resources for authors of reports of randomized trials: harnessing the wisdom of authors, editors, and readers

**DOI:** 10.1186/1745-6215-12-98

**Published:** 2011-04-19

**Authors:** David Moher, Sally Hopewell, Kenneth F Schulz, Douglas G Altman

**Affiliations:** 1 Ottawa Methods Centre, Clinical Epidemiology Program, Ottawa Hospital Research Institute, USA; 2 Centre for Statistics in Medicine, University of Oxford, Wolfson College, Oxford, OX2 6UD, UK; 3 Quantitative Sciences, FHI, North Carolina, USA

## Abstract

The CONSORT Statement was developed to help authors improve the quality of reporting randomized trials. To augment the statement we published the CONSORT explanation and elaboration paper which included at least one example of good reporting for each CONSORT checklist item. We are developing a comprehensive database of examples of good reporting for each checklist item to take advantage of the breadth and variety of trials familiar to authors and readers globally. We invite authors, editors, and readers worldwide to nominate examples of well reported items for the database.

## 

Randomized trials are critically important to healthcare, widely regarded as providing the most reliable evidence on the benefits and harms of interventions. Reports of many randomized trials, most of which are published in specialty and subspecialty journals, have crucial information missing or reported in such a manner that the information cannot be understood and/or used by readers [1]. While improvements have been seen over time, the majority of trials still do not report essential information available from every trial such as full details of the interventions and outcomes, and the methods of allocation of interventions. Glasziou and colleagues reported that only two thirds of the 55 reports of randomized trials they examined provided sufficient details about the interventions such that clinicians could use them in practice [2]. Hopewell and colleagues examined reports of 616 trials published in December 2006 and noted that only 34% described how the randomization sequence was generated and only 25% described an adequate method of allocation concealment; despite improvement since 2000 both remain well under 50% [1]. These examples focus on internal validity. Reporting of issues pertaining to external validity is equally discouraging, if not worse [3,4]. One consequence of unclear trial reports is their potential exclusion from systematic reviews. Clearly, the importance of including this critical information is not appreciated by authors, peer reviewers, or editors.

The CONSORT Statement was developed to help authors improve reporting their randomized trials. CONSORT was first published in 1996 and updated, initially in 2001 and most recently in March 2010 [5]. A systematic review indicated that journal endorsement of CONSORT is associated with improvements in reporting of randomized trials [6].

An innovative feature of CONSORT 2001 was the development of the CONSORT explanation and elaboration (E&E) paper, also updated in 2010 [7]. A distinctive feature of the E&E paper was inclusion of at least one example of good reporting for each of the checklist items. For example, Appendix 1 shows examples in the E&E of good reporting of trial interventions and sequence generation. The inclusion of an example in the E&E indicates that for that trial this specific item was reported well (but it does not imply that the entire article was necessarily a good trial report).

Readers of a report of an RCT need to know how the trial was carried out - clear and complete reporting reveals essential details of trial design and conduct. That information may sometimes reveal some aspects of the trial that were less than ideal. Likewise, readers need a clear presentation of the trial's main findings, in line with the trial protocol. Good reporting provides readers with the information they need to judge whether such weaknesses affect the reliability of the trial findings.

Selecting the examples included in the updated CONSORT 2010 E&E document was a time consuming process, as our goal was to identify examples of both good reporting and good methodology. Moreover, we could not take full advantage of the breadth and variety of published randomized trials familiar to authors and readers globally. To enhance this resource for authors of randomized trial reports, we intend to develop an extensive, searchable, comprehensive database, accessible on the CONSORT website http://www.consort-statement.org, of examples of good reporting for each CONSORT checklist item (e.g., 6a, Outcomes). In order to achieve this, the CONSORT Group is inviting authors, editors, and readers worldwide to nominate examples from published reports of trials of well reported items to be considered for inclusion in the database. Our initial focus is on good reports of sound conduct, as in the E&E paper.

Examples of good reporting should be submitted via e-mail to the CONSORT Examples Library web page http://www.consort-statement.org/consort-library of the CONSORT website. Along with the example submissions, authors will be asked to include their name and e-mail address for correspondence, if we need clarification. The only specific criterion for selecting examples is the consensus view that the example is a good report of the item in question. We do not anticipate corresponding with authors, editors, or readers of items included in the database or those items not selected; this would be time consuming given the very limited resources available to the CONSORT group. We plan to include a list of contributors in the CONSORT database. We hope that this expanded database of examples will further help authors in complying with the CONSORT 2010 Statement as they write up their trial findings for publication. We look forward to hearing from you.

## Appendix 1

Examples of good reporting for two items in the CONSORT checklist

CONSORT item 6: "The interventions for each group with sufficient details to allow replication, including how and when they were actually administered"

Example of a good description of an intervention:

"Patients were randomly assigned to receive a custom-made neoprene splint to be worn at night or to usual care. The splint was a rigid rest orthosis recommended for use only at night. It covered the base of the thumb and the thenar eminence but not the wrist [picture shown]. Splints were made by 3 trained occupational therapists, who adjusted the splint for each patient so that the first web could be opened and the thumb placed in opposition with the first long finger. Patients were encouraged to contact the occupational therapist if they felt that the splint needed adjustment, pain increased while wearing the splint, or they had adverse effects (such as skin erosion). Because no treatment can be considered the gold standard in this situation, patients in the control and intervention groups received usual care at the discretion of their physician (general practitioner or rheumatologist). We decided not to use a placebo because, to our knowledge, no placebo for splinting has achieved successful blinding of patients, as recommended" [8].

CONSORT item 8a "Method used to generate the random allocation sequence"

Example of a good description of sequence generation for simple randomization:

"Independent pharmacists dispensed either active or placebo inhalers according to a computer generated randomisation list" [9].

More complex types of randomization exist, such as blocked randomization, although we did not include examples in the CONSORT 2010 E&E paper.

## Competing interests

The authors declare that they have no competing interests.

## Authors' contributions

DM wrote the initial draft. SH, KFS, and DGA provided feedback. All authors read and approved the final manuscript.
